# The broiler chicken as a model for studying partial exocrine pancreatic insufficiency

**DOI:** 10.1016/j.psj.2026.108066

**Published:** 2026-10-31

**Authors:** V. Vertiprakhov, K. Pierzynowska, S. Pierzynowski, A. Kralinina, S. Karamushkina

**Affiliations:** a The Animal Nutritional Physiology Laboratory, Russian State Agrarian University - Moscow Timiryazev Agricultural Academy, Moscow, 127434, Russian Federation; b Department of Biology, Lund University, Lund, 22332, Sweden; c Anara AB, Trelleborg, 23132, Sweden; d Department of Animal Physiology, The Kielanowski Institute of Animal Physiology and Nutrition, Polish Academy of Sciences, Jabłonna, 05110, Poland

**Keywords:** Pancreas, Enzyme efficacy screening, Animal models, Preclinical studies

## Abstract

A reliable partial exocrine pancreatic insufficiency (pEPI) model does not exist in research practice. All such models used in preclinical and nutritional experimental studies are based on the resection of part of the pancreatic parenchyma. In the current study we attempted to develop a reliable pEPI model, considering the anatomy of the pancreas in the chicken. In the chicken, pancreatic juice enters the duodenum via 3 independent ducts. In the current study we ligated the ventral pancreatic duct, thus developing a pEPI. We have used twelve chickens, which were divided in three groups: the Control group of fistulated chickens, the PDL group of pancreatic duct ligated (PDL) birds and the PDL+Enzymes group, where PDL chickens received porcine pancreatic enzymes. The development of pEPI was confirmed by significant changes in the growth pattern of the chickens, altered profiles of postprandial pancreatic enzyme activity in duodenal chyme and blood, as well as postprandial levels of the end-products of proteolysis in plasma and structural changes in pancreatic tissue of chickens following pancreatic duct ligation. Mixed-effects analysis revealed significant effects of prandial status, pancreatic status and their interactions on trypsin and alkaline phosphatase activity in duodenal chyme. The activity of plasma alkaline phosphatase, as well as phosphatase-protease ratio and total plasma calcium content in PDL birds were significantly decreased by 55%, 63% and 65.7%, respectively, when compared to the Control group. Plasma triglycerides levels in PDL birds were increased by 4.4 fold when compared to the healthy Control birds. All these changes were reactive and sensitive to enzyme supplementation and all the above-mentioned parameters were improved in the PDL+Enzymes group. Due to the fast metabolism of broiler chickens, the current model could be considered as a new, promising tool for the investigation of pancreatic development and pathology, as well as the consequences of EPI with regards to the homeostasis of entire organisms. This model deserves attention and future studies to demonstrate its benefits both for basic science and clinical practice.

**Implications**

The novel chicken model of pEPI is sensitive, responsive and relevant for investigations concerning chicken productivity and humans’ health. Due to the fast metabolism of broiler chickens, the model could be considered as a new, promising tool for the investigation of pancreatic development and pathology, as well as the consequences of EPI for the metabolic homeostasis of entire organisms, screening of new pancreatic enzyme replacement therapy (PERT) alternatives and dose-finding studies. This model undoubtedly deserves attention and future studies to demonstrate its benefits both for basic science, broiler production and clinical practice.

## Introduction

Animal models have been used for the thorough investigation of pancreatic development and pathology, creating a holistic ‘background picture’ corresponding to exocrine pancreatic function and dysfunction and allowing for the accurate assessment of the effectiveness of pancreatic enzymes replacement therapy (PERT), as well as various dietary components in correcting pancreatic function.

Various methods exist for studying the development of the pancreas, the pathogenesis of pancreatic dysfunction and the development of exocrine pancreatic insufficiency (EPI) in different animals. Previous studies have attempted to explore the pathophysiology of chronic pancreatitis in cats, dogs (Churg and Richter, 1971), rats, mice and even the American opossum (*Didelphis virginiana*) (Lerch et al., 1993; Rünzi et al., 1995). A short comparison of pancreatic structural features is provided in Table 1.

**Table 1:** A comparative summary of pancreas anatomy and islet architecture between species.

Species	Gross Form / Shape	Ductal Anatomy	Islet Architecture & Cell Distribution	Key References
Chicken (Avian)	Formed by 3-4 distinct, elongated lobes tucked tightly within the duodenal loop.	Has 2 to 3 main pancreatic ducts draining independently into the distal duodenum.	Dimorphic Islets: Segregated into 'Dark Islets' (predominantly alpha/delta cells) and 'Light Islets' (beta cells).	Hellman and Hellerström, 1960
Human	Compact, solid retroperitoneal organ categorized into a head, neck, body, and tail.	Single main duct (Wirsung) joins the common bile duct at the ampulla of Vater.	Intermingled / Mosaic: Alpha and beta cells are distributed evenly and randomly throughout the islet profile.	Cabrera et al., 2006; Cavallero et al., 2009
Porcine	Tri-lobed, compact mass that wraps around the portal vein, forming a 'portal ring'.	Inverse dominance: The accessory pancreatic duct acts as the primary drainage route.	Intermingled: Mosaic cellular architecture highly comparable to humans.	Ferrer et al., 2008
Rodent (Rat)	Diffuse, dendritic, and soft; dispersed loosely within the mesentery.	Multiple small hepatic ducts merge with pancreatic ducts into a common biliopancreatic duct.	'Core-Mantle' pattern: Beta cells sit in a dense central core; alpha cells encircle the periphery.	Tsuchitani et al., 2016; Baetens et al., 1978
Canine	Bilobed, inverted V-shape or L-shape nestled inside the mesoduodenum.	Dual ducts: A minor pancreatic duct and a larger, patent accessory duct opening distally.	Lobe-Dependent: Left/body lobes have standard configurations, but the right lobe completely lacks alpha cells.	Tsuchitani et al., 2016; Gersell et al., 1979.
Opossum	Compact, V-shaped or L-shaped organ tucked alongside the stomach and duodenum.	A single common biliopancreatic duct that enters near the stomach/duodenal junction.	Core-Mantle: Beta cells cluster in the center with alpha/delta cells along the outer border.	Tsuchitani et al., 2016; Lerch et al., 1993

Despite vast evolutionary distances, chickens (avian) and humans (mammalian) share fundamental physiological similarities in their pancreatic development, cell lineages, and core digestive mechanisms (Tsuchitani et al., 2016). Embryologically, the pancreas in both species develops from the fusion of dorsal and ventral primordial buds arising from the primitive gut endoderm (Tsuchitani et al., 2016). Functionally, human and chicken acinar tissue synthesizes the exact same classes of crucial exocrine digestive enzymes—specifically amylase, lipase, and proteases (trypsinogen)—to break down macronutrients within the small intestine (Vertiprakhov et al., 2024; Pandol, 2010). Furthermore, the final intestinal phase of pancreatic secretion in both organisms is regulated by the highly conserved neurohumoral hormones secretin and cholecystokinin (CCK), which stimulate fluid neutralization and enzyme deployment in response to acidic chyme entering the duodenum (Vertiprakhov et al., 2024; Pandol, 2010).

Undoubtedly, different species develop features of physiological pancreatic dysfunction or chronic pancreatitis differently and to various extents. For example, mammalian pancreatic secretion is down-regulated during suckling (Pierzynowski et al., 1995), most likely due to the presence of enzymes in the mother’s milk. When working with animal models, other differences are noted, for instance dogs slowly develop mild changes in pancreatic parenchyma, while rats and mice demonstrate initial changes including pancreatic oedema and inflammatory cell infiltration, consistent with acute pancreatitis (Watanabe et al., 1995) following pancreatic duct ligation (PDL). Whereas, signs of chronic pancreatitis, such as atrophy, acinar cell loss, and fibrosis develop as soon as two weeks after PDL surgery (Aghdassi et al., 2011). Moreover, the rodent pancreas is diffuse, soft, and dendritic, scattered as a microscopic, web-like meshwork throughout the thin mesentery (Tsuchitani et al., 2016), while chickens possess a well-defined lobular organ (Hellman and Hellerström, 1960; Vertiprakhov et al., 2024). At the same time, in rodents, the multiple tiny pancreatic ducts fuse completely with the hepatic bile ducts to form a common biliopancreatic channel before entering the intestine (Tsuchitani et al., 2016). Surgical PDL in a rodent inadvertently blocks bile flow, causing confounding liver failure and jaundice (Tsuchitani et al., 2016). The chicken possesses 2 to 3 completely independent primary pancreatic ducts routing away from bile channels directly into the duodenum, allowing surgeons to tie off specific pancreatic conduits to study isolated exocrine atrophy, without blocking bile mechanics (Vertiprakhov et al., 2024). Exocrine pancreatic flow drops to near-zero levels in rodents during fasting to conserve fluid (Pandol, 2010). Avian species maintain high, continuous basal secretion even during prolonged starvation, yielding roughly 25 mL of active pancreatic juice daily from a single chicken (Vertiprakhov et al., 2024). This provides an abundant biological fluid pool for downstream fractional proteomics and enzyme kinetics tracking, without the need to pool animals.

All the above-mentioned features of rodents’ nutritional physiology and anatomy of the gastrointestinal tract, as well as their nutritional requirements and feeding regimen, make them an inappropriate model for translational studies, since the relevance of findings in the field of exocrine pancreatic function for human clinical practice and/or other species is comparatively low.

As mentioned, a series of intriguing results have been obtained from studies using the American opossum (*Didelphis virginiana*). The anatomy of the pancreas in these animals resembles that of the human pancreas and the surgical ligation of the common pancreatic and bile duct at the papilla of Vater can lead to the development of acute necrotizing pancreatitis (Lerch et al., 1993; Rünzi et al., 1995). However, this model in general has not been well unexplored and the use of this species in research is very limited. Currently, the domestic pig (*Sus scrofa domesticus)*, with surgically induced exocrine pancreatic insufficiency, represents a very well-established model of chronic pancreatitis with changes in pancreatic morphology appearing as early as two weeks after PDL (Nothman et al., 1948; Gewert et al., 2004; Gregory et al., 2016). This model is highly relevant and well investigated, however it undoubtedly has its limitations, including the high cost of the animals and their maintenance. Moreover, since this model is highly relative to humans, the testing of treatment efficacy often requires long-term studies, which makes the pig model undesirable for fast screening.

A summary of available animal models for the study of EPI is provided in Table 2.

**Table 2:** Comparison of available animal models of exocrine pancreatic insufficiency (EPI).

Species & Model Type	Primary Induction Method	Degree of EPI & Islet Preservation	Potential Issues & Limitations	Relevance for Humans	Key References
Canine • Spontaneous	Natural genetic Pancreatic Acinar Atrophy (PAA).	Complete / Severe. Total loss of acinar tissue. Islets fully intact (no diabetes).	Spontaneous onset limits timing flexibility. High cost and companion-animal ethical barriers.	Gold standard for human clinical idiopathic EPI. Perfectly mirrors pathobiology and steatorrhea without surgical artifacts.	Wiberg, 2004; Westermarck and Wiberg, 2003
Porcine • Surgical (PDL)	Ligation of the Accessory Pancreatic Duct.	Severe & Stable in roughly 80% of operated animals. Good islet survival early; late fibrosis risk.	Anatomical Variations: ∼20% fail due to undetected accessory micro-duct safety valves or main pancreatic duct maintenance. High cost	Premier model for translational testing of Pancreatic Enzyme Replacement Therapy (PERT) due to high digestive tract and body-weight homology.	Nothman et al., 1948; Gewert et al., 2004; Gregory et al., 2016; Luu et al., 2020; Schubert et al., 2022
Rodent (Rat/Mouse) • Dietary	Copper-deficient diet combined with D-penicillamine.	Severe & Selective. Massive synchronous acinar cell apoptosis. Islets remain unharmed.	Systemic Toxicity: Causes anemia, cardiomegaly, and vascular issues. Spontaneous Recovery: Trace copper triggers rapid regeneration.	Ideal for high-throughput therapeutic screening and analyzing molecular pathways of exocrine tissue regeneration.	Fell et al., 1982; Rao et al., 1993
Rodent (Rat/Mouse) • Surgical	90% to 95% surgical resection (partial pancreatectomy) leaving a 10% duodenal remnant.	Variable. Operator-dependent due to the diffuse nature of the tissue. Poor islet preservation; extreme risk of secondary diabetes.	Highly meticulous surgery due to diffuse anatomy. Confounding diabetic pathologies mask pure malabsorption studies. High immediate post-op mortality.	Directly models post-pancreatectomy human clinical states (such as post-Whipple procedures or near-total pancreatectomy) and the onset of Type 3c diabetes mellitus secondary to tissue ablation.	Brockenbrough et al., 1988; Bonner-Weir et al., 1993.
Rodent (Rat/Mouse) • Chemical-Obstructive	Intraductal retrograde infusion of a viscous zein solution (corn protein) to cause mechanical occlusion.	Severe & Irreversible. Induces rapid acinar pressure, permanent block, and severe lobular atrophy. Good islet survival; long-term diffuse fibrosis over 6 months.	Technically challenging surgical cannulation of the tiny rodent duct. Can induce localized acute chemical inflammation if infusion pressure is not controlled.	Simulates complete clinical exocrine failure without removing the islets or inflicting systemic mineral deficiencies to evaluate downstream changes in hormone pathways.	Tomita et al., 1988; Dubick et al., 1989
Chicken (Avian) • Dietary	Severe, targeted selenium deficiency.	Moderate to Severe. Reversible upon selenium replacement. Endocrine functions largely spared.	• Significant evolutionary gap in digestion rates. • Selenium deficiency creates a systemic 'dirty' phenotype.	Used strictly for specialized studies regarding cellular antioxidant mechanisms and nutritional tissue remodeling pathways.	Noguchi et al., 1973

Since pancreatic dysfunction remains a problem and investigation of the resulting pancreatic secretion pathology remains relevant, we propose (in the current study) a new method for the development of an animal model of partial EPI (pEPI) that has shown promising results and is expected to help us better understand the pathogenetic and etiological basis of chronic pancreatitis. Moreover, use of the current model, together with our recently developed technique for the measurement of di- and tripeptide absorption, could highlight the contemporary question as to why mammalian growth is so extremely variable between individuals.

The aim of the present, explorative study, was to describe a new animal model which could be more cost-efficient and at the same time appropriate for the screening of new dietary and curative treatment strategies. Changes in body weight gain patterns and duodenal chyme enzyme activity, which are dependent on prandial status and pancreatic morphology, were considered as primary model outcomes/primary objectives, while the changes in duodenal chyme mineral content, proteolysis efficacy and blood parameters were considered as secondary objectives. We developed a broiler chicken model of PDL, which has the advantage of not only ligating the pancreatic duct, but also inserting a cannula into the duodenum opposite the confluence of the pancreatic and bile ducts. This allowed us to obtain duodenal chyme and study the activity of the pancreatic enzymes in it.

## Materials and methods

All manipulations on birds were performed in accordance with the requirements of the humane treatment of animals (European Convention for the Protection of Vertebrate Animals used for Experimental and other Scientific Purposes (ETS No. 123) (Strasbourg 18.03.1986). The studies were approved by the Bioethics Committee of the Russian State Agrarian University - Moscow Agricultural Academy named after K.A. Timiryazev (approval number 46 05.05.2025). All efforts were made to minimize animal suffering.

### Animals and care

The study was conducted on twelve broiler chickens of ROSS-308 cross strain, aged 15 days, with a body weight of 766.5±214.4 g at the start of the study (prior to surgery). Animals were divided into three groups each consisting of four chickens. The first group served as a control group and animals had an implanted duodenal fistula (implanted into the duodenum in the section of the intestine opposite the confluence of the pancreatic ducts (Vertiprakhov, 2022); the second group of birds (PDL) had a duodenal fistula and a ligated ventral pancreatic duct to disrupt the outflow of pancreatic juice and impair the secretory function of the pancreas; the third group of birds (PDL+Enzymes) had a duodenal fistula and a ligated ventral pancreatic duct and were supplemented with an enzyme preparation made from pig pancreatic tissue with a trypsin activity of 187 U/ml, added to their feed at a dose of 100 mg/kg. The enzyme preparation was prepared using porcine pancreas lyophilization and contained active lipase, protease, and amylase and the dosage was chosen based on a previous study by our lab which made use of the same preparation (Vertiprakhov et al., 2025). The chickens were assigned to groups using a simple randomization method, with the aid of an online random number generator. All chickens came from an initial healthy pool of same-age birds, with strict balancing for sex (2 pullets and 2 cockerels in each group). The ventral pancreatic duct ligation surgery was performed under the same surgical conditions as the insertion of the duodenal fistula, which have been thoroughly described by (Vertiprakhov, 2022).

Broiler chickens were fed complete ROST feed throughout the entire experimental period (Tosnensky Mixed Feed Factory, Nurma, Leningrad Region, Russian Federation, feed composition and recipe compounds are provided in Table 3, Table 4, respectively). Chickens were individually housed in cages allowing for olfactory and visual contact, with *ad libitum* access to water. Light regimen was set according to the recommendations for this chicken cross (18 h of light and 6 h of darkness). To assess the health status and secretory function of the pancreas in the broiler chickens, experiments began 3-5 days after surgery.

**Table 3:** Composition of the ROST feed.

Parameter	Value
Feed weight	1 kg
Moisture	9.81 %
ME poultry WPSA	310 kcal
ME poultry VNIIKP	315.67 kcal
Crude protein	19.44 %
Crude fiber	5.14 %
Crude fat	9.13 %
Crude ash	5.47 %
Lysine	1.11 %
Methionine	0.53 %
Methionine + Cystine	0.86 %
Threonine	0.78 %
Tryptophan	0.24 %
Arginine	1.26 %
Valine	0.88 %
Isoleucine	0.78 %
Leucine	1.35 %
Glycine	0.85 %
Digestible Lysine	1.02 %
Digestible Methionine	0.51 %
Digestible Methionine + Cystine	0.8 %
Digestible Threonine	0.68 %
Digestible Tryptophan	0.21 %
Digestible Arginine	1.14 %
Digestible Valine	0.76 %
Digestible Isoleucine	0.69 %
Digestible Leucine	1.24 %
Digestible Glycine	0.68 %
Calcium (Ca)	0.8 %
Total Phosphorus (P)	0.73 %
Available Phosphorus	0.4 %
Sodium (Na)	0.15 %
Potassium (K)	0.7 %
Chlorine (Cl)	0.23 %
Linoleic acid	5.55 %
Linolenic acid	0.06 %

ME - Metabolizable Energy; WPSA – World’s Poultry Science Assosiation; VNIIKP - All-Russian Research Institute of the Feed Industry, Abbreviations used for the specific standardized nutritional calculation methodologies used in poultry feed formulation.

**Table 4:** ROST feed compounds.

Raw Material	%
Wheat 11.5%	42.64
Soybean meal 46%	15.38
Sunflower meal 36%	15.38
Corn 8.5%	15.00
Sunflower oil	7.41
Limestone Ca 36%	1.24
Monocalcium phosphate	1.21
Lysine sulfate	0.58
Premix 0.5%	0.50
Salt	0.32
Methionine	0.21
Threonine	0.13
**Total**	**100.00**

### Experimental flow

Experiments began daily in the morning, with the collection of duodenal chyme after a 14-h fast. The birds were then fed 30% of their daily feed intake, and the duodenal chyme collection was repeated 60 min after feeding. After the second chyme collection birds had *ad libitum* access to their standard feed. Feed consumption was controlled during the entire study period and was 180 g per bird per day. Control over possible leftovers was performed daily. Duodenal chyme sample volume was between 0.5-1.0 ml. Body weight measurements were made weekly during the experimental period, which lasted for thirty days.

### Blood sample collection

Blood samples were collected from the axillary vein prior to and 60 min after feeding. Samples were transferred to glass tubes coated with K_2_EDTA. The tubes were immediately placed on ice before they were centrifuged at 3000 × g for 15 min at 4°C, and plasma was separated and stored at −80°C until further analysis.

### Plasma and duodenal chyme samples analysis

Plasma biochemical analysis was done using the BioChem FC-120 (High Technology, Inc, USA) analyzer. Alkaline phosphatase activity, as well as calcium and phosphorus content in plasma and duodenal chyme were measured using the BioChem SA (High Technology, Inc, USA) analyzer. Trypsin activity assay was performed using BAPNA as a substrate for proteolytic activity as previously described (Vertiprakhov et al., 2018).

### Estimation of the level of nutritional peptides

Pre- and postprandial levels of nutritional peptides in plasma were assessed using the modified ninhydrin method in plasma samples, according to Pierzynowska et al. (2024), Pierzynowski et al. (2025).

### Autopsy

At the end of the experimental period animals were euthanized using an intravenous infusion of xylazine hydrochloride (Ksilanit, Nita-Farm, Saratov, Russian Federation), at a dose of 10 mg/kg bwt.

### Histological assessment of pancreatic tissue

On autopsy, according to examination with the naked eye, pancreata were not inflamed and were collected and fixed in 10% neutral buffered formalin for 24 h. Then, the specimens were dehydrated and embedded in paraffin according to standard histological techniques using the АГТ-11 FMP (Phizmedpribor-M LLC, Moscow, Russian Federation) tissue processor. Paraffin-embedded tissues were cut into 1.5-3-micrometer-thick sections using a sledge microtome МС-1 PA (NPK Orion Medic LLC, St. Petersburg, Russian Federation). After drying overnight, the slides were deparaffinized by incubation in xylene and decreasing concentrations of ethanol. Sections were used for standard hematoxylin and eosin staining to estimate parameters such as fibrosis, acinar cells necrosis, eosinophilic infiltration, duct obstruction, acini atrophy. The grades for each parameter scoring are shown in Table 5. The analysis was performed using a light microscope (Hariomed IW-A1350C SN24/1007 (Hua Yue Medical Technology Co., Ltd., Guangzhou, China) equipped with a digital camera (ADF live 4k, ADF Optics, Guangzhou, China). The obtained data were assessed using ADF software version 3.2 (ADF Optics, Guangzhou, China). A minimum of 20 measurements of each parameter were made for each section. The measurements were averaged by section, and after that the sections were averaged per bird. The histological analysis was blinded (personnel were unaware of treatment and/or group allocations).

**Table 5:** Histological assessment of pancreatic injury.

Category/Parameter Score	Score
Fibrosis	0 = <5% 1 = 5-10%
	2 = 10-25%
	3 = >25%
Acinar Cells Necrosis	0 = absent 1 =few necrotic cells /<5% of 400x field 2 = multiple necrotic cells /5-20% of 400x field
	3 = large necrotic areas/>20% of 400x field
Eosinophilic Infiltration	0 = none
	1 = 1-5 eosinophils per 400x field
	2 = 6-20 eosinophils per 400x field 3 = >20 eosinophils per 400x field
Duct Obstruction	0 = normal ducts, open lumen 1 = mild: single ducts with narrowed lumen 2 = moderate: most intralobular ducts are deserted or have epithelial atrophy. 3 = severe: almost all ducts are obliterated or replaced by fibrosis.
Acini Atrophy	0 =Absent: normal acini, normal size and density 1 = Mild: Slight decrease in acini size, slight parenchymal rarefaction 2 = Moderate: Noticeable decrease in the size and number of acini, increased distance between them, beginning of fibrosis 3 = Severe: Severe atrophy, acini are greatly reduced or almost absent, most of the parenchyma is replaced by fibrous tissue

### Statistical analysis

To evaluate body weight trajectories while controlling for the nested structure of repeated measures, data were analyzed using a Bayesian Linear Mixed Model (LMM). The model specified Treatment (Control, PDL, PDL+Enzymes), Time (Weeks 0, 2, 3, and 4), and their two-way interaction (TreatmentXTime) as fixed effects. To account for the statistical dependency of longitudinal measurements within individual birds and capture baseline weight variations, animal ID was modeled as a random effect providing by-subject random intercepts.

Analysis was performed using JASP (version 0.98.1, Amsterdam, the Netherlands), utilizing Markov Chain Monte Carlo (MCMC) sampling with default Jeffreys-Zellner-Siow (JZS) priors. Chain convergence and sampling efficiency were verified using the Potential Scale Reduction Factor (R̂ ≤ 1.003) and Effective Sample Size parameters (Bulk-ESS and Tail-ESS > 2000). Fixed effects structure and overall treatment differences were evaluated via Estimated Marginal Means and pairwise post-hoc contrasts. Trajectory variations and interactions were interpreted using posterior estimate means alongside their corresponding 95% highest posterior density (HPD) and credible intervals (CI). Effects were deemed statistically supported if their 95% CI or HPD entirely excluded zero.

Statistical analysis of data collected on other study parameters was performed using GraphPad Prism 11.0.2 (GraphPad Software LLC., San-Diego, USA). The data distribution was assessed using the Shapiro–Wilk normality test. Normally distributed data was analyzed using either the Brown-Forsythe and Welch ANOVA or Welch’s t-test for three or two groups, respectively. Data with nonparametric distribution was analyzed using either the Kruskal-Wallis or Mann-Whitney test for three or two groups, respectively. A mixed effects analysis was conducted to estimate the effects of prandial and pancreatic status on duodenal chyme parameters.

Since no sample size estimation was done *a priori*, the achieved statistical power was estimated *post-hoc* using G∗Power software, version 3.1.9.4, for the respective type of analysis, using data generated in GraphPad Prism.

Data was not corrected for multiple comparisons due to the explorative nature of the study and the inclusion of planning for the measurable outcomes of the study design (Rothman, 1990; Althouse, 2016). Differences were considered significant if p ≤ 0.05; differences were considered as a trend when p ≤ 0.1 (0.05 ≤ p ≤ 0.10); normally distributed data is expressed as mean ± standard deviation (SD), data with nonparametric distribution is shown as median ± interquartile range (IQR).

## Results

### Body weight gain patterns

Body weight gain trajectories of the chickens are shown in Fig. 1. A Bayesian Linear Mixed model was applied to evaluate chicken body weight patterns across 48 observations nested within 12 individual birds. The model reached excellent MCMC convergence stability (R̂ ≤ 1.003; bulk/tail ESS > 2000), establishing fit baseline metrics at Watanabe-Akaike Information Criterion (WAIC) = 749.5 (SE = 13.36) and Leave-One-Out Cross-Validation (LOO) = 753.5 (SE = 14.76). Partitioning subject-specific variation revealed an ID random intercept variance of (5.383*10^4^) (σ= 232.0) against a residual variance of (2.374*10^5^) (σ= 487.3), yielding an Intraclass Correlation Coefficient (ICC) of 0.1848.

**Fig. 1:**
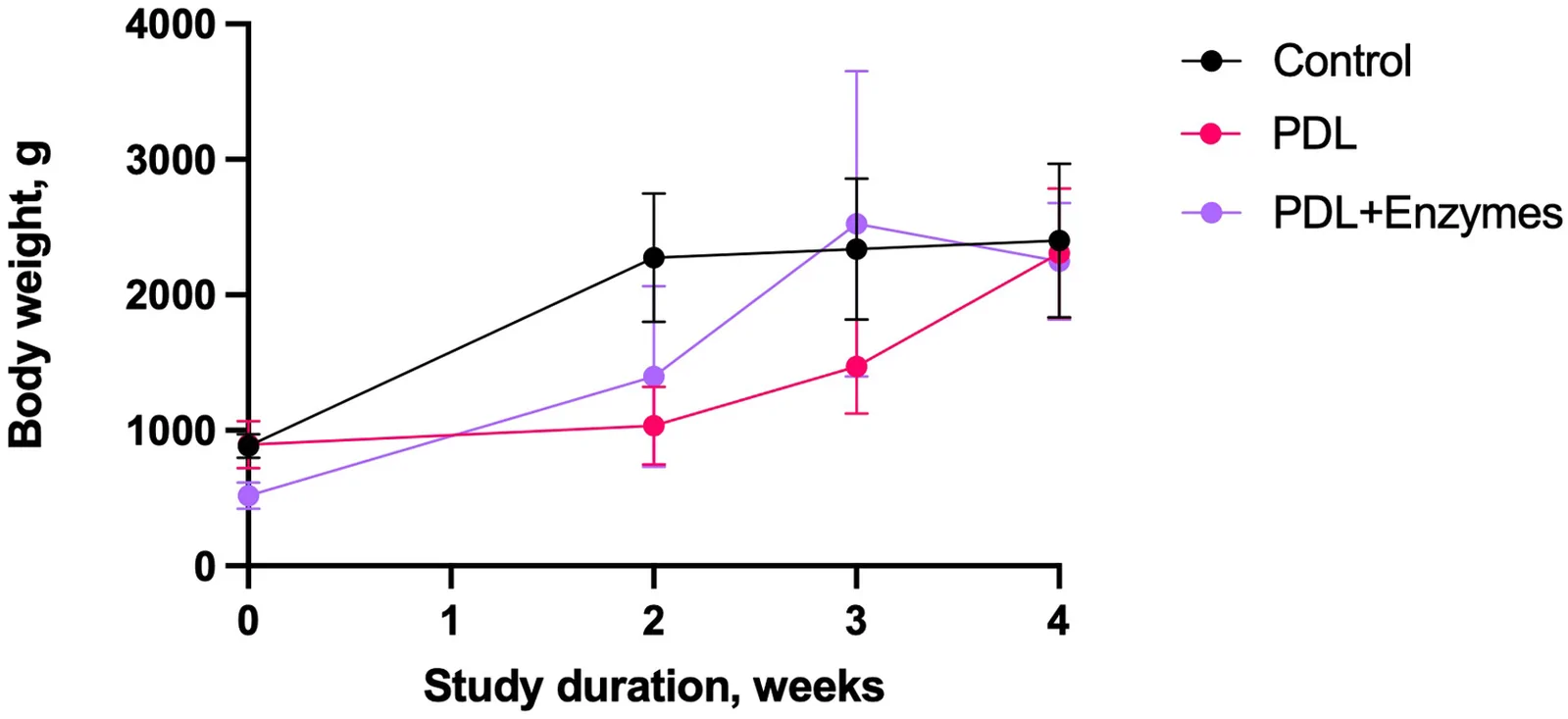
**Body weight changes in experimental chickens.** The control group - chickens with an implanted duodenal fistula (n=4); the PDL group – chickens with a duodenal fistula and a ligated ventral pancreatic duct (n=4). Data is presented as Mean ±SD, points are connected with solid lines. Dashed lines represent the linear regression analysis outcome.

Highly credible standalone main effects were confirmed for both Pancreatic Status (Control: 282.6, 95% CI: [6.308, 557.1]; PDL: -263.4, 95% CI: [-533.6, -5.914]) and Time.

Evaluation of the Pancreatic Status X Time interaction estimates exposed highly distinct, non-parallel growth trajectories among the three groups across the four-week timeframe. The Control group established a trajectory characterized by a massive initial growth velocity. By Week 2, the Control group displayed an independent positive upward deviation from the model baseline (Estimate = 582.9, 95% CI [73.80, 1085.0]), continuing steady growth to peak at Week 4 (Estimate = 710.5, 95% CI [206.0, 1223.0]). The PDL group trajectory was defined by severe, localized growth stunting during the first half of the study. At Week 2, the PDL group demonstrated a profound negative trajectory interaction (Estimate = -654.6, 95% CI [-1171.0, -145.0]). This developmental delay persisted heavily into Week 3 (Estimate = -217.7). However, between Weeks 3 and 4, the LMM captured a significant, late-phase compensatory growth surge (Estimate = 615.5, 95% CI [100.5, 1113.0]), which allowed the group to catch up to the Control weight envelope by the time the study was terminated. The addition of enzymes completely reshaped the early stunting function associated with the PDL group (following PDL surgery). While the cohort tracked closely with the restricted groups through Week 2 (Estimate = -293.7), the LMM isolated an explosive, highly supported mid-study acceleration inflection point unique to this group at Week 3 (Estimate = 835.2, 95% CI [356.9, 1321.0]). This sudden trajectory escalation successfully bypassed the extended stunting phase observed in the PDL group, bringing the animals into full recovery an entire week ahead of the unsupplemented PDL group.

Post-hoc contrast analysis, utilizing 95% HPD intervals, isolated a single highly credible pairwise group difference between Control and PDL groups, confirming that healthy control birds maintained significantly superior body weight tracks compared to the untreated pancreatic duct ligated birds (Estimate: 547.6, 95% HPD: [83.49, 1015]). Pairwise differences for Control vs. PDL+Enzymes and PDL vs. PDL+Enzymes encompassed zero, indicating that while enzyme supplementation mathematically shifted median weights upward, it did not push them to a level statistically distinct from either boundary group.

### Duodenal chyme parameters

For the estimation of changes in pancreatic secretion, the duodenal chyme samples, collected both pre-prandially and 60 min after feeding, were analyzed for trypsin and alkaline phosphatase activity, as well as for calcium and phosphorus content.

Chickens in both the Control and PDL+Enzymes groups had similar pre-prandial trypsin activity, which increased significantly (p<0.05) after feeding by 40% and 36%, respectively (Table 6). Chickens that underwent PDL without external enzyme supplementation (PDL group) displayed raised pre-prandial trypsin activity in the duodenal chyme, which significantly decreased by 19% after feeding (Table 6). According to the mixed-effects model analysis, trypsin activity was significantly affected by prandial status (F (1.98) = 6.688, p = 0.0112), but the influence of pancreatic status failed to reach significance. However, the interaction between pancreatic status and prandial status also played a significant role in determining trypsin activity (F (1.837, 124.9) = 12.25, p ≤0.0001).

**Table 6:** Enzyme activities and mineral content in the duodenal chyme of experimental chickens.

Parameter	Control		PDL		PDL+Enzymes	
	fasted	fed	fasted	fed	fasted	fed
Trypsin activity, U/L	1364±505^ac^	1920±966^b^	1719±668^a^	1399±572^c^	1364±451^c^	1860±741^b^
Alkaline phosphatase activity, U/L	16426±2997^b^	13036±6464^ac^	10282±5815^a^	11549±13232^abd^	15406±4055^c^	11194±5005^d^
Total Calcium, mM	12.48±11.34^a^	16.52±15.36^abc^	17.84±7.74^b^	17.48±6.30^abc^	13.99±7.66^ab^	21.42±10.32^c^
Phosphorus, mM	5.63±1.14^a^	5.93±0.87^a^	5.69±0.89^a^	3.77±0.74^b^	5.84±1.04^a^	6.13±1.18^a^

The control group - chickens with an implanted duodenal fistula (n=4); the PDL group – chickens with a duodenal fistula and a ligated ventral pancreatic duct (n=4); the PDL+Enzymes group – chickens with a duodenal fistula and a ligated ventral pancreatic duct, supplemented with an enzyme preparation made from pig pancreatic tissue, with a trypsin activity of 187 U/ml, added to their feed at a dose of 100 mg/. Data is presented as Mean ±SD, different small letters given with the results indicate a significant difference between means within a row at p<0.05.

Alkaline phosphatase activity was similar in chickens in both the Control and PDL+Enzymes groups pre-prandially and decreased significantly 60 min after feeding by 21% and 26%, respectively (Table 6). Pre-prandial alkaline phosphatase activity in PDL birds was not different from that observed in chickens in the Control group and the values remained unchanged after feeding (Table 6). The mixed-effects model analysis demonstrated that alkaline phosphatase activity was not affected by prandial status, but was significantly influenced by both pancreatic status (F (1.658, 164.2) = 64.448, p = 0.0186) and the interaction between prandial and pancreatic status (F (1.658, 164.2) = 5.979, p ≤0.0053).

The highest pre-prandial duodenal chyme total calcium content was observed in chickens in the PDL group (43% higher when compared to control birds). Total calcium content in duodenal chyme was not significantly altered by feeding in both Control and PDL chickens. However, it is worth noticing that postprandial levels of total calcium in duodenal chyme were 53% higher than pre-prandial levels in chickens in the PDL+Enzymes group (Table 6). The mixed-effects model analysis revealed a significant effect of prandial status on total calcium content in duodenal chyme (F (1, 62) =4.86, p=0.0312).

Pre- and postprandial phosphorus content in duodenal chyme were similar between experimental groups, except for a significant 34% postprandial drop in observed in PDL chickens (Table 6). The mixed-effects model analysis demonstrated that phosphorus content was not affected by prandial status but was significantly influenced by both pancreatic status (F (1.900, 34.21) = 9.883, p = 0.0005) and the interaction between prandial and pancreatic condition (F (1.900, 34.21) = 9.641, p ≤0.0006).

No significant effects of random effect (animal) were observed for any of the investigated parameters.

The post-hoc computation of statistical power for the duodenal chyme parameters showed that for the trypsin activity the observed main effect of Prandial State achieved a power of 73.7%, while its interaction with pancreatic status achieved 99.3% power. Analysis of alkaline phosphatase activity achieved powers of 5.2% for the negligible effect of Prandial Status, 67.9% for the effect of Pancreatic Status, and 80.9% for their interaction. For total calcium content, the observed effects yielded powers of 58.3% for Prandial Status, 35.7% for Pancreatic Status, and 40.1% for their interaction. Phosphorus content demonstrated strong statistical support, achieving 46.9% power for the main effect of Prandial Status, 96.6% for Pancreatic status, and 96.1% for their interaction.

### Analysis of proteolysis efficacy and its end-product absorption

To investigate whether PDL and the resulting altered pattern of trypsin activity fluctuations in the duodenum could influence proteolysis, we analysed proteolysis efficacy and the profiling of absorbed proteolysis end products. Pre-prandial levels of total amine group equivalents were 239.1±145.1 μg/ml in healthy, control chickens, which significantly increased by 55%, 60 min after feeding (Fig. 2A). Pre-prandial levels of total amine group equivalents were unaffected (161.9±40.3 μg/ml) by feeding in PDL chickens (Fig. 2A). At the same time, profiling of proteolysis end products in plasma revealed that the plasma content of free amino acids remained unchanged after feeding in healthy, control chickens, but the proportion of dipeptides significantly increased postprandially, while there was a tendency for an increase in the proportion of tripeptides (Fig. 2B). The pre-prandial proportion of free amino acids was highest in control chickens and this parameter significantly changed after PDL surgery (Fig. 2B). The post-hoc computation of statistical power for the proteolysis efficacy showed that the achieved power reached 99.1%.

**Fig. 2:**
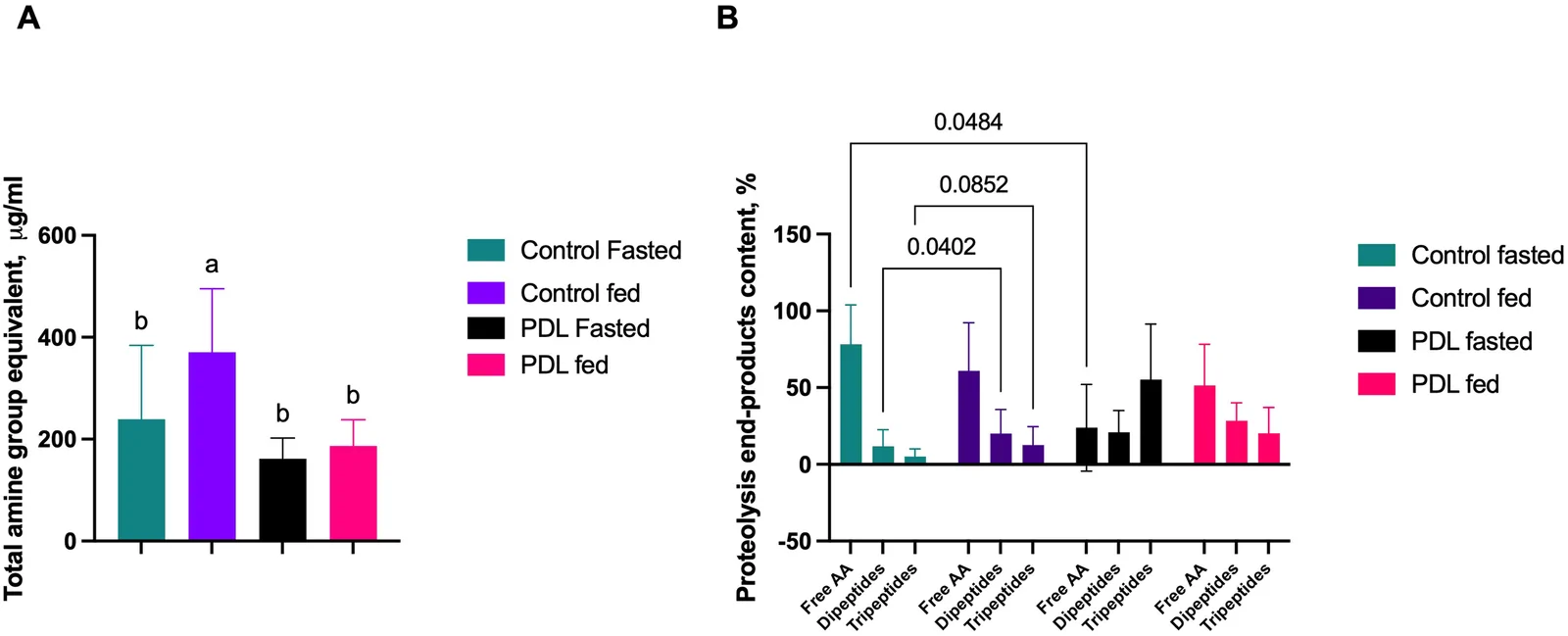
**Total amine group equivalents (A) and profile of proteolysis end-product absorption (B) in the plasma of experimental chickens.** The control group - chickens with an implanted duodenal fistula (n=4); the PDL group – chickens with a duodenal fistula and a ligated ventral pancreatic duct (n=4). Data is presented as Mean ±SD. Different small letters given with the result bars indicate a difference in means at p<0.05.

### Main biochemical parameters in circulation

To investigate how pancreatic duct ligation affects the systemic levels of enzyme activities, metabolites and minerals, the collected blood samples were analyzed. Blood trypsin and amylase activity, as well as cholesterol and phosphorus content was similar between groups, but alkaline phosphatase activity was significantly decreased in PDL chickens (by 55% when compared to healthy, control chickens, p<0.05) and this decline was reversed by enzyme supplementation (no significant differences in alkaline phosphatase activity were observed between Control and PDL+Enzymes groups) (Table 7). A similar pattern with regards to changes and reversibility by enzymatic supplementation was observed for parameters such as Phosphatase-Protease Ratio and total calcium content, which were significantly decreased by 63% and 65.7%, respectively in chickens in the PDL group (Table 7).

**Table 7:** Enzyme activities, metabolites and mineral content in the plasma of experimental chickens.

Parameter	Group			Power Estimates	
	Control	PDL	PDL+Enzymes	Cohen's f	Achieved Power (1 - β)
Trypsin activity, U/L	84.93±17.42	97.50±16.47	83.77±13.65	15.5446	**>99.9%**
Amylase activity, U/L	669.3±173.9	552.3±76.7	463.0±176.7	1.6448	**99.20%**
Alkaline phosphatase activity, U/L	780.8±247.0^a^	356.0±176.8^b^	869.3±151.0^a^	1.5481	**98.40%**
Phosphatase-Protease ratio	9.3±3.0^a^	3.5±1.8^b^	11.3±2.9^a^	1.2608	**91.50%**
Glucose, mM	9.2±1.3^a^	11.7±0.9^b^	12.8±1.8^b^	1.144	**85.30%**
Total Protein, g/L	42.9±5.1^a^	32.0±3.0^b^	30.3±2.0^b^	1.089	**81.50%**
Uric Acid, μM	96.3±48.6^a^	187.7±46.3^b^	177.8±72.5^ab^	0.7179	45.30%
Triglycerydes, mM	0.15±0.07^a^	0.66±0.26^b^	0.08±0.04^a^	0.5638	29.70%
Cholesterol, mM	5.3±0.9	4.7±0.5	5.5±0.5	0.5144	25.30%
Total Calcium, mM	2.65±0.05^a^	0.91±0.05^b^	2.70±0.06^a^	0.3903	16.20%
Phosphorus, mM	2.04±0.24	1.95±0.07	2.04±0.13	0.2608	9.70%

The Control group - chickens with an implanted duodenal fistula (n=4); the PDL group – chickens with a duodenal fistula and a ligated ventral pancreatic duct (n=4); the PDL+Enzymes group – chickens with a duodenal fistula and a ligated ventral pancreatic duct, supplemented with an enzyme preparation made from pig pancreatic tissue with a trypsin activity of 187 U/ml, added to feed at a dose of 100 mg/kg. Data is presented as Mean ±SD, different small letters given with the results indicate a significant difference between means within a row at p<0.05.

Interestingly, blood glucose concentrations in chickens in the PDL and PDL+Enzymes groups were higher by 26.6% and 38.5%, respectively, when compared to that observed in the Control Group (Table 7). In contrast, blood total protein levels in chickens from the PDL and PDL+Enzymes groups were reduced by 25.5% and 29.5%, respectively (p<0.05) in comparison to that of the Control group chickens and this change was irreversible, irrespective of enzyme supplementation (Table 7).

Uric acid levels were significantly increased following pancreatic duct ligation (by 94.9% in the PDL group when compared to the Control Group. Enzyme supplementation in the PDL+Enzymes group failed to significantly improve blood uric acid levels and no significant differences were observed between PDL and PDL+Enzymes groups (Table 7). At the same time, blood triglyceride levels of chickens in the PDL group were significantly increased (by 4.4-fold) when compared to Control chickens, but this gain was successfully reversed by enzyme supplementation, and no differences were observed between chickens in the Control and PDL+Enzymes groups (Table 7). Results of the post-hoc statistical power analysis are also presented in Table 7.

### Blood morphological analysis

Analysis of the blood cellular component count revealed that PDL chickens had significantly (p<0.05) decreased leucocytes (32%), lymphocytes (14%), leucocytes/heterophils ratio (16%) and hemoglobin content (26%) when compared to the healthy Control birds (Table 8). The above-mentioned decreases were accompanied by significant increases in the number of eosinophils (by 46%) and heterophils (Mann-Whitney p=0.0252, actual difference between medians is 1.5, Hodges-Lehmann difference is 2.0) (Table 8). Results of the post-hoc statistical power analysis are also presented in Table 8.

**Table 8:** Blood cellular components count.

Parameter	Group			
	Control	PDL	Cohen's d	Achieved Power (1 - β)
Leucocytes count, x10ˆ9/L	34.0±3.9	23.0±2.8*	3.2402	**96.50%**
Lymphocytes, %	54.8±2.7	48.8±1.2*	2.8718	**91.90%**
Heterophils, %**#**	25.0±2.0	26.5±9.0*	2.4666	**82.60%**
Lymphocytes/Heterophils Ratio	2.23±0.1	1.84±0.2*	2.2265	74.70%
Basophils,%**#**	5.0±1.0	6.0±3.0	2.1891	73.30%
Eosinophils,%	9.3±2.5	13.6±1.1*	1.0647	24.60%
Monocytes,%**#**	6.0±4.0	5.5±2.0	0.4506	8.40%
Erytrocytes, x10ˆ12/L	2.6±0.29	2.5±0.12	0.4472	8.40%
Haemoglobin, g/L	105.0±17.2	78.0±2.9*	0.2301	5.90%
Haematocrit,%	32.0±3.8	29.0±1.2	0.1581	5.40%

The Control group - chickens with an implanted duodenal fistula (n=4); the PDL group – chickens with a duodenal fistula and a ligated ventral pancreatic duct (n=4). Data is presented as Mean ±SD, # - data is presented as Median ± IQR, * given with the results indicates a difference between groups at p<0.05.

### Morphological analysis of pancreatic tissue

Histological investigation revealed that four weeks after ventral PDL surgery, various pathological pancreatic changes were observed including fibrosis, acinar cell necrosis, eosinophilic infiltration, duct obstruction and acini atrophy. Some mild changes were observed in the pancreata of chickens in the healthy, Control group of chickens, however, the total histological score analysis revealed a significant (p<0.001) difference between Control and PDL groups (both actual difference and Hodges-Lehmann difference between medians were 4.0) (Fig. 3). Post-hoc computation of statistical power showed that achieved power reached 99.9%.

**Fig. 3:**
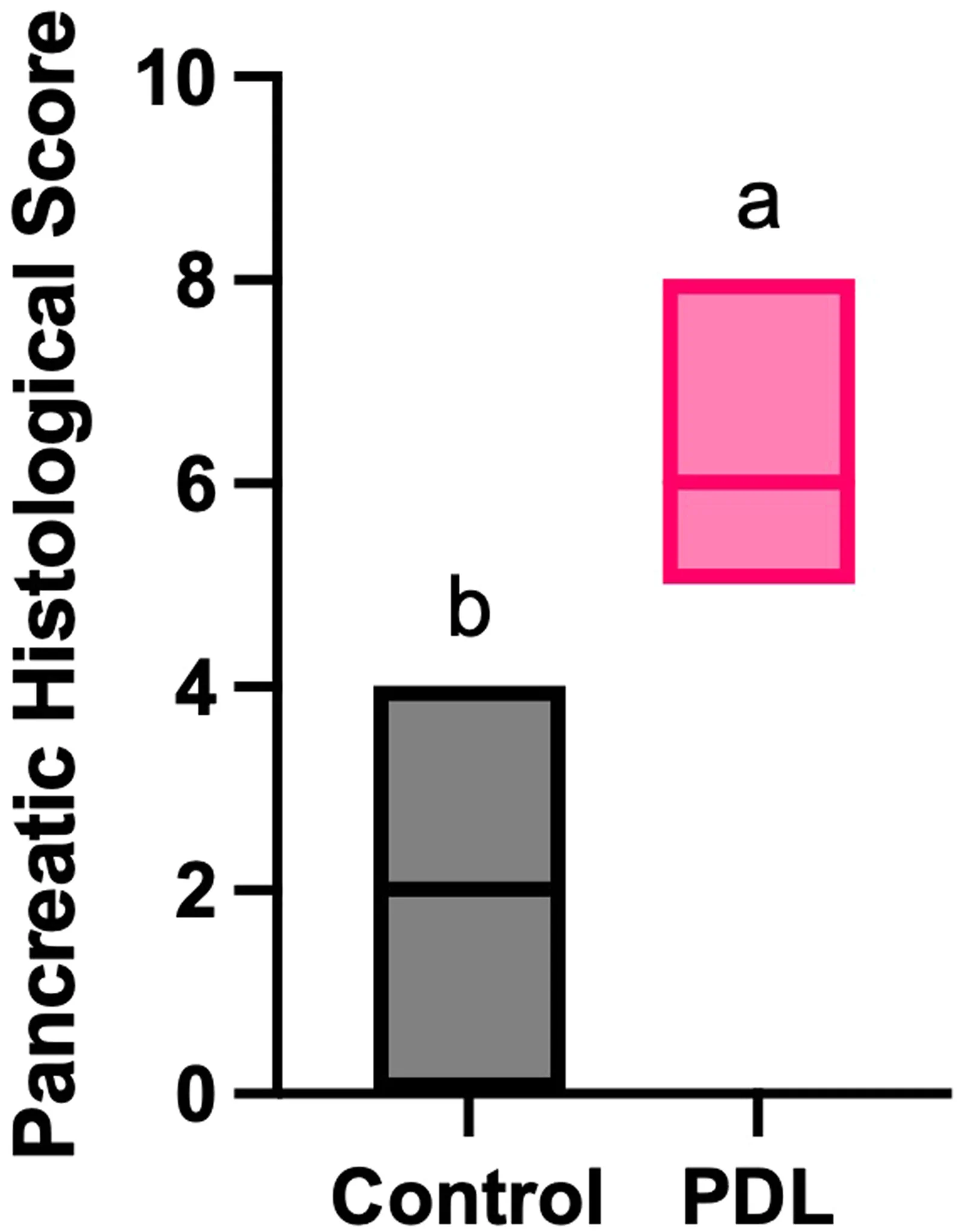
**Total pancreatic histological score.** The control group - chickens with an implanted duodenal fistula (n=4); the PDL group – chickens with a duodenal fistula and a ligated ventral pancreatic duct (n=4). Data is presented with a line at the Median. Small letters given with the result bars indicate a difference in means at p<0.05.

## Discussion

Exocrine pancreatic insufficiency (EPI) often appears as a consequence of a disease, such as cystic fibrosis, acute or chronic pancreatitis, pancreatic cancer, diabetes, etc., but it can also be age-related. E.g., all suckling mammals exhibit a peri-natal pancreatic insufficiency in comparison to their pancreas function after weaning (Pierzynowski et al., 1995). Thus, it is worth mentioning that mother’s milk is actually the ideal food, the digestion of which requires very minimal energy expenditure. Of course, the enzymatic component of fresh, mother’s milk can’t be neglected. Consequently, studies on pancreatic expenditure during yolk development in the chicken, in comparison to that during milk consumption in mammals would be very interesting. The general prevalence of EPI in the human population is estimated to be between 10-20%, but at the same time EPI is considered to be “an under screened, underdiagnosed and untreated” pathology (Lewis, 2024). Pancreatic enzyme replacement therapy (PERT) has remained the only form of treatment available to EPI patients, for more than 100 years. Unfortunately, neither the optimization of PERT doses, nor the use of alternative, non-animal sources of enzymes have been found to be very successful thus far in completely alleviating EPI symptoms. Thus, the need for additional EPI research, the discovery of new and customization of already existing animal models, is obvious.

It is very important to note that unlike rats and rabbits, whose pancreas is comprised of diffuse glandular tissue spread across the mesentery, the pancreas in birds (as in humans, pigs and dogs) is a compact, well-defined, discrete, lobulated organ. The relative mass of the organ in broilers (0.19–0.53%) is comparable to that in pigs (0.11–0.15%) and humans (0.1–0.2%) (Batoev, 2001). This allows for the successful application of the unique Batoev-Vertiprakhov fistula technique for the in *vivo* continuous sampling of duodenal chyme, which is physically unfeasible with the diffuse glandular tissue of small rodents. Moreover, both complex-reflex and chemical phases of pancreatic secretion in chickens are fundamentally identical to those in mammals (Pandol, 2010; Vertiprakhov et al., 2024).

The monthly weight gain in broiler chickens can reach as much as 2 kg. The 30-day monitoring period of the present study covers >70% of the lifespan of a broiler chicken (∼45 days), enabling the study of genuine chronic tissue changes (such as atrophy or fibrosis). According to the laws of Kleiber and van 't Hoff, the combination of mass-specific metabolic rate R=M^-1/4^; a 2.43-fold acceleration) and the kinetics associated with a high body temperature (41.5°C; a 1.40-fold acceleration) yields an integral biological acceleration coefficient of 3.42. Thus, 30 days in a broiler are mathematically equivalent to more than 100 days (∼3.5 months) of continuous pathological progression in a human. Thus, we assumed that this animal model should be vulnerable and sensitive to possible disturbances in pancreatic function. In 2001, Batoev et al. described the ventral pancreatic duct in chickens as being the most highly developed, since it collects pancreatic juice from the main mass of the pancreas. The dorsal pancreatic duct receives secretions from the anterior portion of the pancreas, on the superior side of the mesentery. Between the ducts of the ventral and dorsal lobes, there is also a small duct collecting pancreatic juice from a small area of the gland adjacent to the cranial end of the ventral lobe. Thus, we performed the procedure of ligation using the ventral pancreatic duct. It is important to note that our attempts to ligate all three pancreatic ducts, or both the dorsal and ventral pancreatic ducts, led to the development of severe acute pancreatitis and animal death – within 24 h after surgery. The duodenal port insertion surgery, which is a widely used technique in our lab, allowed us to obtain duodenal chyme samples, which could be used for the estimation of pancreatic enzyme activity, both pre- and postprandially. The PDL surgery led to the development of partial, compensated pancreatic insufficiency (pEPI), which was quantitatively verified using the following anatomical and biochemical criteria: (1) The avian pancreas possesses three independent ducts. The ligated ventral duct is the primary duct which drains both the ventral and splenic lobes, which in turn account for 40–50% of the total pancreas mass. The dorsal and intermediate ducts remained intact following ligation of the ventral duct. (2) Direct *in vivo* measurements showed that, at the postprandial peak (at 60 min after feeding), trypsin activity in the chyme of PDL chickens decreased by 32.5% compared to that observed in the Control group, rather than falling to zero. This demonstrates that the two remaining ducts, through compensatory hypersecretion, retain 67.5% of the pancreatic proteolytic capacity, thereby preventing lethal pancreatic necrosis in the bird.

It is important to acknowledge the lack of baseline measurements, which could have been performed after fistula insertion prior to the PDL surgery. We decided to perform both surgical procedures within the same surgery in order to minimize animal suffering, according to the 3R principle. A sufficient pool of data on duodenal chyme enzyme activity and blood parameters exists for broiler chickens with a duodenal fistula (Hulan et al., 1972; Bohn et al., 2018; Sergeenkova et al., 2024a, 2024b; Vertiprakhov, 2022)

The first important finding of our study is the difference in growth patterns of the chickens. The growth arrest, which is a characteristic feature of EPI in children, has been previously observed in different large animal models of EPI, including naturally, surgically- and chemically induced models (Beliaev, 1994; Fedkiv et al., 2009; Lebenthal and Lee, 1982). Beliaev (1994) observed a significant decrease in body weight following EPI development and its restoration by PERT supplementation, in a canine model of trypsin-induced EPI; Fedkiv et al. (2009) showed that pancreatic enzymes are crucial for growth regulation in young pigs (Fedkiv et al., 2009), but do not affect body weight gain in grower finisher pigs. Our current findings revealed a significant difference between the trajectories of body weight gain observed in healthy Control chickens and PDL chickens and showed that body weight gain trajectory can be restored by enzyme supplementation, as evidenced in the PDL+Enzymes group. These observations are in line with both previous animal studies and clinical data, which show the sensitivity and relevance of the broiler chicken pEPI model.

The composition of duodenal chyme directly reflects pancreatic status and sampling the duodenal chyme makes observations during both the post- and pre-prandial stages of pancreatic secretion possible. In the current study we showed that in PDL chickens the pattern of enzyme secretion, both for trypsin and alkaline phosphatase, is dysregulated and cannot support nutrient digestion. Thus, the basal trypsin activity in PDL chickens is much higher when compared to Control chickens and it drops at 60 min after feeding, in this way confirming a disturbance of the complex reflex regulations of pancreatic function. One should note that high basal trypsin activity is not recognized as being beneficial, since protease in the absence of substrate can be harmful for the duodenal mucosa and thus could lead to an inflammatory process, as well as fibrosis, etc. of the duodenum. Our results confirm the importance of PERT supplementation, since it was clearly shown that enzyme administration normalized both the basal trypsin levels, and its’ activity at 60 min after feeding.

To investigate how disturbances in protease secretion and release would affect proteolysis and the absorption of the proteolysis end-products, we measured total amine group equivalents and analyzed the proteolysis end-product plasma profile. We clearly showed that in control, healthy chickens the low pre-prandial level of total amine group equivalents is drastically increased after feeding, thus reflecting sufficient protease action; while in PDL chickens, with a disturbed trypsin pre- and postprandial activity, the pre- and post-prandial levels of total amine group equivalents were similar and the values were comparable to basal levels observed in healthy chickens. This observation is in line with previously observed data from a porcine model of EPI (Pierzynowska et al., 2025), as well as data from clinical studies (Sathe et al., 2025). Moreover, the plasma proteolysis end-products absorption profile revealed that healthy birds absorb proteins mainly in the form of di- and tripeptides, as the proportion of these protein derivatives was increased after feeding. Taking into consideration that the postprandial plasma profiling of model organisms requires more profound investigation, it is clear that the measure of amine group equivalents is sensitive to protease activity and could be used on a routine basis for the investigation of the effectiveness of pancreatic enzymes.

In general, many plasma parameters appeared to be sensitive to PDL and could be used for the assessment of EPI status. Despite the lack of significant change in either plasma trypsin or amylase activity between groups, the significant changes observed in alkaline phosphatase activity and phosphatase/protease ratio reflect the insufficiency of the exocrine pancreas. In birds, intestinal alkaline phosphatase is a vital brush-border enzyme linked to nutrient absorption (especially lipids and calcium), mucosal defense, and the regulation of duodenal surface pH. Because its expression and activity are highly dynamic and responsive to luminal nutrients (Hanauska et al., 2025), our results on alkaline phosphatase activity both in duodenal chyme and plasma, which were in both cases sensitive to enzyme supplementation, highlight a major breakdown in normal postprandial signaling caused by EPI.

Pancreatic enzyme supplementation restored plasma alkaline phosphatase activity and the phosphatase/protease ratio to levels observed in healthy chickens, which confirms the sensitivity of these two parameters and their importance in this novel chicken model of pEPI. Interestingly, blood total protein levels were decreased after PDL. This change was irreversible and could not be corrected by pancreatic enzyme supplementation, which is in line with the observed changes in growth patterns and brings up new questions regarding EPI pathogenesis and consequences for growth and lean body mass decline.

Irreversible, PDL-stimulated changes, have also been observed for other plasma parameters, such as glucose and uric acid. The increase in plasma glucose levels observed in the current study is in line with previous observations in a porcine model of EPI (Pierzynowska et al., 2023; Pierzynowska et al., 2018; Pierzynowski et al., 2017, 2018) and could be hypothetically explained by the disturbances in the acini-islet-acinar axis, which is ultimately regulated by pancreatic amylase. The almost 2-fold increase in plasma uric acid content was a very interesting finding. Elevated plasma levels of uric acid in cystic fibrosis patients have been observed in clinics and were associated with high doses of pancreatic extracts used as PERT (Nousia-Arvanitakis et al., 1977). The elevated levels of plasma uric acid observed in the current study cannot be explained by the enzyme supplementation, as this increase doesn’t develop exclusively under porcine enzyme supplementation, but is rather a consequence of PDL. To date, our existing pool of knowledge does not allow for a detailed explanation of the mechanisms of action involved, however the increased plasma uric acid content could be hypothetically explained by the EPI-stimulated disturbances in liver (Patel et al., 2022) or renal function (Zaworski et al., 2026). Another possible explanation could be the trypsin deficiency in the chyme, which induced protein malabsorption. Given the broiler's hyper-intense metabolism, this nutrient deficit could trigger the compensatory breakdown of the bird's own muscle proteins to sustain energy pathways. The accelerated breakdown of endogenous proteins could in turn result in an excessive release of nitrogenous waste products, manifesting as the consistent spike in plasma uric acid levels observed in the PDL group.

Two more plasma parameters, which appeared to be sensitive to pancreatic status, were triglycerides and calcium content. Plasma triglyceride concentration in pEPI chickens was elevated, while plasma calcium content decreased. Both these changes were very sensitive to enzyme supplementation. Since we measured the total calcium content, one could couple the decrease in this parameter to the observed decrease in the total plasma protein levels, as approximately 50% of the total calcium plasma content is protein-bound (Schenck and Chew, 2008). However, the reactivity of calcium content to the enzyme supplementation at the time, when this supplementation failed to restore total protein levels, allows us to hypothesize that this enzyme-reactive pool of calcium is rather not protein-bound. Taking into consideration all the above mentioned changes, one could suggest that the lack of sufficient pancreatic function could easily lead to metabolic disturbances, such as elevated glucose, uric acid and triglycerides levels. The decreased ability to digest and absorb proteins, as well as the altered growth pattern observed in the chickens, confirm this assumption.

Blood morphological analysis demonstrated that PDL results in a decreased number of almost all blood cell types, except for eosinophils and erythrocytes. The erythrocyte count remained unchanged following PDL, despite the drop in hemoglobin levels, but for a thorough investigation, one should probably monitor the fate of the red blood cells for their entire lifespan (more than 90 days).

The number of eosinophils increased following PDL, and the resulting pancreatic inflammation was the obvious reason for this observation. Histological analysis of pancreatic tissue revealed a strong eosinophilic infiltration, pancreatic ducts obstruction, necrosis of acinar cells and fibrosis. These observations are in line with the clinical pathologists’ observation and findings from larger animal models (canine and porcine models of EPI) and confirm a similar mechanism of pEPI development in this novel chicken model as that in previously established animal models and in patients suffering from EPI.

While this exploratory study provides promising insights into the model, certain limitations must be addressed. Although the sample size (n = 4 per group) and the study duration of one month were sufficient for an initial proof-of-concept, larger cohorts and longer studies are needed to confirm the generalizability of these results. Additionally, future studies should include a sham-operated group to control for surgical stress, as well as healthy animals supplemented with porcine enzymes to determine potential baseline effects. The inclusion of digestibility trials will also be crucial to directly quantify nutrient absorption. The inability to measure all parameters in animals receiving pancreatic enzyme supplementation represents another limitation of our work, as well as the lack of the time effect estimation and thus, the incapacity to follow the pEPI development. Nevertheless, despite these limitations, the current findings offer a strong foundation for using this broiler chicken model in pancreatic research and all the prior mentioned limitations are subjects of further studies and give directions for future experiments. Despite the small sample size, majority of measurements were adequately powered and achieved statistical power of at least 80%. At the same time, the effect sizes observed for all the parameters investigated allow us to appropriately plan for future studies. Our plans for further studies include, but are not limited to, the profound analysis of glucose metabolism parameters, such as levels of insulin, glucagon and incretins and the biochemical assessment of hepatic and renal function, including hepatic transaminases activity, urea and creatinine levels, etc.

Thus, despite all the mentioned limitations, our explorative study has shown that the broiler chicken model of pEPI induces a complex phenotype of secondary exocrine insufficiency developing as a result of mechanical ductal occlusion. Of course, the model doesn’t allow us to distinguish between “pure” EPI effects and the effects of pancreatic tissue fibrosis and inflammation, however, it successfully reaches the universal terminal phenotype of EPI, which includes acinocyte necrosis, reactive stromal fibrosis, and a luminal enzyme deficiency. Due to the fast metabolism of broiler chickens, the current model could be considered as a new, promising tool for the investigation of pancreatic development and pathology, as well as the consequences of EPI with regards to the homeostasis of entire organisms and the screening of new PERT alternatives and dose-finding studies. This model undoubtedly deserves attention and further validation in terms of nutrient digestibility, renal and hepatic function investigation, as well as detailed examination of developmental mechanisms to verify its possible relevance for other species and possible benefits both for basic science and clinical practice.

## Author Contribution Statement

V. Vertiprakhov: developed the study design, obtained funding and drafted the initial version of the manuscript; S. Pierzynowski: Conceived the study concept, acquired and interpreted obtained data, drafted the initial version of the manuscript and critically reviewed all versions of the manuscript prior to submission; K. Pierzynowska: acquired, analyzed and interpreted data, drafted the initial version of the manuscript and critically reviewed all versions of the manuscript prior to submission; A. Kralinina: acquired, analyzed and interpreted data, critically reviewed all versions of the manuscript prior to submission; S. Karamushkina: acquired, analyzed and interpreted data, critically reviewed all versions of the manuscript prior to submission. All authors have read and approved the final version of the manuscript and agree to be personally and publicly responsible for all aspects of the work.

## Ethics approval

The studies were approved by the Bioethics Committee of the Russian State Agrarian University - Moscow Agricultural Academy named after K.A. Timiryazev (approval number 46 05.05.2025).

## Data Availability Statement

The raw data supporting the conclusions of this article will be made available by the authors upon request.

## Declaration of Generative AI and AI-assisted technologies in the writing process

The authors did not use any artificial intelligence assisted technologies in the writing process.

## Financial support statement

This study was conducted by the authors without external support.

## Disclosures

The authors of this manuscript have the following competing interests: Stefan Pierzynowski and Kateryna Pierzynowska are the owners of Anara AB, Sweden. All other authors declare no competing interests.
